# Can Animal Models Contribute to Understanding Tinnitus Heterogeneity in Humans?

**DOI:** 10.3389/fnagi.2016.00265

**Published:** 2016-11-14

**Authors:** Jos J. Eggermont

**Affiliations:** ^1^Department of Physiology and Pharmacology, University of Calgary, CalgaryAB, Canada; ^2^Department of Psychology, University of Calgary, CalgaryAB, Canada

**Keywords:** brain imaging, neural responses, neural synchrony, spontaneous activity, burst firing, human, animal

## Abstract

The brain activity of humans with tinnitus of various etiologies is typically studied with electro- and magneto-encephalography and functional magnetic resonance imaging-based imaging techniques. Consequently, they measure population responses and mostly from the neocortex. The latter also underlies changes in neural networks that may be attributed to tinnitus. However, factors not strictly related to tinnitus such as hearing loss and hyperacusis, as well as other co-occurring disorders play a prominent role in these changes. Different types of tinnitus can often not be resolved with these brain-imaging techniques. In animal models of putative behavioral signs of tinnitus, neural activity ranging from auditory nerve to auditory cortex, is studied largely by single unit recordings, augmented by local field potentials (LFPs), and the neural correlates of tinnitus are mainly based on spontaneous neural activity, such as spontaneous firing rates and pair-wise spontaneous spike-firing correlations. Neural correlates of hyperacusis rely on measurement of stimulus-evoked activity and are measured as increased driven firing rates and LFP amplitudes. Connectivity studies would rely on correlated neural activity between pairs of neurons or LFP amplitudes, but are only recently explored. In animal models of tinnitus, only two etiologies are extensively studied; tinnitus evoked by salicylate application and by noise exposure. It appears that they have quite different neural biomarkers. The unanswered question then is: does this different etiology also result in different tinnitus?

## Tinnitus Heterogeneity

One may classify tinnitus types by etiology, phenotype, comorbidity or all these combined, and personal responses to it (Møller, 2011; Kreuzer et al., 2014). Within the etiology one may distinguish noise trauma and ototoxic drugs, whiplash and neck trauma, blast- and other traumatic brain injury, vestibular schwannoma and Ménière’s disease, and stress. Phenotype differences such as tinnitus pitch, loudness, and aurality may be important as well, but estimates of pitch and loudness are varying between tests (Hoare et al., 2014). Comorbidities of the neurological type such as migraine or tension-type headaches (Langguth et al., 2015), psychological type, such as depression and distress or finding tinnitus bothersome (Schecklmann et al., 2013; Pattyn et al., 2016), and of the audiological type such as hyperacusis (Schecklmann et al., 2014) seem to be more important for treatment than etiology. Moreover, these comorbidities together with the amount of hearing loss appear to underlie most of the electro- and magneto-encephalography (EEG/MEG) and brain imaging findings, whereas tinnitus on its own barely affects these (Davies et al., 2014). Here it should be emphasized that in animal experiments one knows the etiology, knows typically exactly what structures, subdivisions and neuron types one is recording from and assumes that optionally resulting stress has no effect. Yet, behavioral test often show that not all animals subjected to a tinnitus-inducing agent will have tinnitus.

What is important from the point of view of animal experiments is how to translate tinnitus types, if they can be solidified, into animal models. Different etiologies that have been studied are noise trauma, ototoxic drugs (i.e., salicylate, quinine, cisplatin), and interaction between somatic stimulation and noise trauma (Eggermont, 2012). In animal research, the only extensive studied etiologies are salicylate application and noise exposure, hence we will compare these two etiologies.

## The Neural Correlates of Salicylate and Noise-Exposure in Animal Models of Tinnitus

### Salicylate

Salicylate induces tinnitus, either following a single high dose (acute) or following repeated administration of low dose (chronic). The result of salicylate application in rodents is predictable and maybe for that reason salicylate has early on been applied in animal experiments (Stypulkowski, 1990; Chen and Jastreboff, 1995; Ochi and Eggermont, 1996). Salicylate interacts with the auditory system in multiple ways in the cochlea and in the central auditory system. In the cochlea, salicylate initially down-regulates the action of prestin in the wall of the outer hair cells (OHCs) and thereby causes a modest hearing loss (Greeson and Raphael, 2009). In addition, salicylate interacts with the arachidonic acid cycle ultimately causing an increase in NMDA receptor activity and increased spontaneous firing rates (SFRs) in a subset of auditory nerve fibers (ANFs; Guitton et al., 2003). Long-duration application reverses its action on prestin and actually enhances its expression (Yu et al., 2008; Yang et al., 2009) and may even lead to ANF degeneration (Deng et al., 2013). Centrally, salicylate down-regulates serotonin and GABA activity, and affects the conductivity of some K^+^ channels (Wang et al., 2008). Cochlear perfusion with salicylate does not produce the central effects of systemically applied salicylate. This makes searching for neural substrates of tinnitus difficult at the least. Salicylate also increases the gain of the more central parts of the auditory system for sound, reflected in increased startle responses and potentially inducing hyperacusis (Sun et al., 2009). So it is not clear what enhanced gap-startle responses after salicylate application imply: tinnitus or hyperacusis (Salloum et al., 2016). This also may depend on the presence or absence of modulation by auditory cortical activity of the gap-startle reflex. As far as SFRs are concerned, high levels of salicylate result in variable changes in ANF, dorsal cochlear nucleus (DCN), inferior colliculus (IC) including central nucleus (ICC) and external cortex (ICX), and auditory cortex (ACx) particularly in primary (A1) and second auditory cortical area (A2), depending on the species, the dose, and type of neuron. An overview is presented in **Table 1**.

**Table 1:** Changes after salicylate application.

Structure	Cell density	SFR	2-DG	Glu	Gly/GABA	5-HT
OHC IHC	≈^19^					
ANF	⇓ (chronic)^18^	≈^4^ ⇑^5^ ⇓^13∗^				
DCN		⇓ (FF)^1^ ≈ (CW)^1^	⇓^15^		⇓^9^	
ICC		⇓^11^⇑^13∗^	⇓^15^ ⇑^16∗^		⇓^8,10, 20^	⇑^17^
ICX		⇑^14^	⇑^15^			
MGB		⇑^13∗^		⇓^7∗^	⇓^7∗^	
A1		≈^2^ ⇓^3∗,6^	⇑^15^			⇑^17^
A2		⇑^12,13∗^	⇑^15^			

*^1^Superfusion in slice FF, fusiform cells; CW, cartwheel cells (Wei et al., 2010); ^2^cat (Ochi and Eggermont, 1996); ^3∗^rat (Yang et al., 2007); ^4^Stypulkowski (1990) (≤200 mg/kg, acute); ^5^Kumagai (1992) (≥400 mg/kg; chronic); ^6^cat (Zhang et al., 2011), ^7∗^Su et al. (2012) slice, ^8^Butt et al. (2016), ^9^Zugaib et al. (2015), ^10^Zou and Shang (2012), ^11^Ma et al. (2006), ^12^Eggermont and Kenmochi (1998), ^13∗^Chen et al. (2015), ^14^Chen and Jastreboff (1995), ^15^Wallhäusser-Franke et al. (1996), ^16∗^Paul et al. (2009), ^17^Caperton and Thompson (2011), ^18^Deng et al. (2013), ^19^Feng et al. (2010), ^20^Bauer et al. (2000), ^∗^ indicates behavioral tinnitus.*

### Noise Trauma

The findings for traumatic noise exposure are summarized in **Table 2**, using the same format as for salicylate. The primary targets of noise trauma (and ototoxic drugs) are the cochlear hair cells. The most vulnerable are the OHCs in the first row followed by the inner hair cells (IHCs). If the noise is not excessively loud and of short duration, the minimal structural damage that correlates with hearing loss is related to changes in the hair cell stereocilia, which contain the transduction channels. If the result of noise exposure is just a temporary threshold shift (TTS), the only consequence may be loss of IHC ribbon synapses followed by permanent loss of the Type I spiral ganglion cells that innervate the IHC (Kujawa and Liberman, 2009). Consequently, central nerve degeneration may ensue. Noise trauma rarely caused increases in SFR of ANFs but more generally a reduction. The result of reduced auditory nerve output is typically an imbalance between neural excitation and inhibition in the central auditory system (Potashner et al., 1997; Llano et al., 2012; Schreiner and Polley, 2014). This causes strong hyperactivity in the DCN (Kaltenbach et al., 2000), and can result in tonotopic map reorganization, likely only in thalamic and cortical areas, accompanied by increased SFR and increased spike-firing synchrony (Noreña and Eggermont, 2003). This trio of changes is considered to comprise potential neural substrates of tinnitus. The balance between the excitatory and inhibitory transmitter efficacy in the central nervous system (CNS) is only temporarily changed in the first few weeks to months after the trauma (Suneja et al., 1998a,b). It is believed that during that period restoration of the excitatory–inhibitory balance can prevent tonotopic map reorganization as well as increases in SFR and neural synchrony, and thus likely also tinnitus (Noreña and Eggermont, 2005). Lesion studies suggest that the DCN may function as a source of increased SFR without ascending cochlear input and descending input from the CNS (Zacharek et al., 2002; Brozoski et al., 2012). However, these studies also suggest that behavioral tinnitus persists in animals for which the DCN output is isolated from central auditory structures. In contrast, the increased SFR in IC is dependent on output of the cochlea (Robertson et al., 2013), at least for the first 8–12 weeks after the trauma (Mulders and Robertson, 2013). This suggests that the induced increased central gain amplifies the remaining SFR from the auditory periphery. If the SFR from the periphery was not amplified the total result would not be an increased SFR in the IC. Species dependence and recovery times may play a role in these discrepancies.

**Table 2:** Changes after chronic NIHL.

Structure	Cell density	SFR	2-DG	Glu	Gly/GABA	5-HT
OHC IHC	⇓^14^					
ANF	⇓^18^	⇓^8^				
VCN		⇑^9^				
DCN	⇓^18^	⇓^1^⇑^2, 12∗^	⇑^3∗^	⇑^16^	⇓^15^	
ICC		⇑^6, 7, 10∗^		⇑^16^	⇓→⇑^16^	⇑^17^
ICX				⇑^16^		
MGB		⇑^4∗^			⇓^5∗^	
A1		⇑^11,13∗^				⇑^17^
A2						

*^1^Fusiform cells (*in vivo*, Ma and Young, 2006), ^2^fusiform cells (slice, Finlayson and Kaltenbach, 2009), cartwheel cells (slice Chang et al., 2002), ^3∗^Middleton et al. (2011) using flavoprotein imaging, ^4∗^Kalappa et al. (2014), ^5∗^Llano et al. (2012) using flavoprotein, ^6^Ma et al. (2006), ^7^Manzoor et al. (2012, 2013), ^8^Liberman and Kiang (1978), ^9^Vogler et al. (2011), ^10∗^Coomber et al. (2014), ^11^Noreña and Eggermont (2006), ^12∗^Brozoski et al. (2002), ^13∗^Basura et al. (2015), ^14^Liberman and Beil (1979), ^15^Potashner et al. (1997), ^16^Suneja et al. (1998a; 1998b), ^17^Caperton and Thompson (2011), ^18^Morest et al. (1998), ^∗^ indicates behavioral tinnitus.*

### Heterogeneity in the Salicylate and Noise Exposure Induced Markers for Tinnitus

**FIGURE 1:**
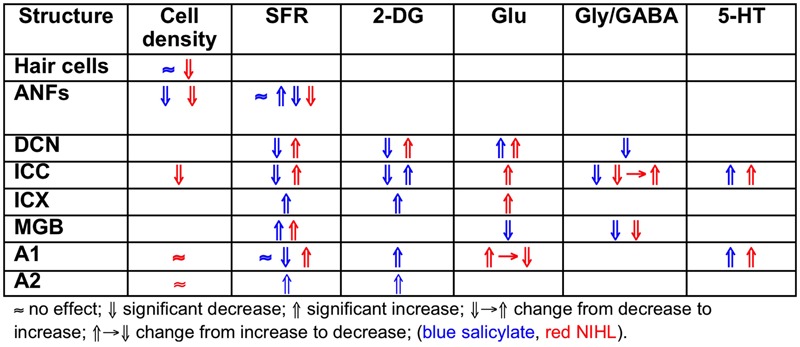
**Comparing the effects of salicylate and noise-induced hearing loss**.

Comparing the findings in salicylate and chronic noise trauma (**Figure 1**) indicates strong differences in SFR and 2-DG, but more correspondence for neurotransmitter action. This is surprising unless we abandon the hypothesis that hyperactivity reflected in increased SFR and 2-DG is a biomarker for tinnitus. In TTS-induced tinnitus, Wu et al. (2016) showed that increased SFRs, burst firing, and spike-firing synchrony in the fusiform cells of the DCN correlated with behavioral evidence for tinnitus. In recordings from cat A1 following salicylate application, Ochi and Eggermont (1996) could not demonstrate an overall change in SFR, however, units that initially had SFRs < 1 sp/s showed a significant increase and units with SFRs > 1 sp/s showed a significant decrease after acute salicylate application. However, Eggermont and Kenmochi (1998) did find a significant increase in SFR in A2 following salicylate application. In neither case could a change in spike-firing synchrony be demonstrated. Noreña and Eggermont (2003) have also shown that immediately after noise exposure, the SFR in A1 was not increased, whereas after more than 2 h it was. In contrast, the spike-firing synchrony was significantly increased immediately after exposure and continued to increase in parallel with the increase in SFR. In the IC, the delay to increased SFR was about 12 h (Mulders and Robertson, 2013), and in the DCN at least 2 days (Kaltenbach et al., 2000). This suggests that the locus of spike recording can result in quite different conclusions if one uses the SFR as a metric.

It is instructive to look at changes in SFR, burst firing, and spike-firing synchrony associated with tinnitus (**Table 3**). Burst firing has been implicated with plastic changes in many neural systems (Eggermont, 2015), and has been evaluated in DCN (Wu et al., 2016), ICC (Bauer et al., 2008; Coomber et al., 2014), and medial geniculate body (MGB; Kalappa et al., 2014) in animals with behaviorally demonstrated putative signs of tinnitus. Increased burst firing correlates strongly with increased SFR in all central areas including ACx. Increased neural spike-firing synchrony, increased bursting and increased SFR correlate in DCN. In recordings from A1 increased spike-firing synchrony is found in the absence of bursting and initially unchanged SFR, but corresponds, after a few hours delay, to increased SFR. This strengthens the idea that increased SFR, at least in subcortical structures, is a biomarker for tinnitus. In salicylate, there is only evidence for bursting and increased SFR in the ICX (Chen and Jastreboff, 1995), but not in the ICC (Ma et al., 2006). In ANFs, bursting only occurs in neurons with very low SFR after noise trauma. This survey suggests that changes in bursting in subcortical structures are not independent of changes in SFR or in spike-firing synchrony. Burst firing and spike-firing synchrony in primary ACx appear to be independent, at least under ketamine anesthesia.

**Table 3:** Burst-firing and Tinnitus.

Structure	Agent	PTS	TTS	SFR	Bursting	Synchrony
ANF	Noise	•		⇓ ≈^1^	⇑^1^	
DCN	Noise	•		⇑^2^	⇑^2^	
	Noise	•		⇑^4∗^	⇑^3^	⇑^4∗^
	Noise		•		⇑^4∗^	
ICC	Noise		•	⇑^5∗^	⇑^5∗^	⇑^5∗^
	Noise	•		⇑^6∗^	⇑^6∗^	
ICC	Noise	•		≈^7^	≈^7^	
	Salicylate		•	⇓^7^	≈^7^	
ICX	Salicylate		•	⇑^8^	⇑^8^	
MGBv	Noise		•	⇑^9∗^	⇑^9∗^	
A1	Noise		•	⇑^10^	⇑ ≈^10^	⇑^10^
A1	Noise	•		⇑^11^	≈^11^	⇑^11^

^1^Liberman and Kiang (1978), ^2^Finlayson and Kaltenbach (2009), ^3^Pilati et al. (2012), ^4^*Wu et al. (2016), ^5∗^Bauer et al. (2008), ^6∗^Coomber et al. (2014), ^7^Ma et al. (2006), ^8^Chen and Jastreboff (1995), ^9∗^Kalappa et al. (2014), ^10^Noreña and Eggermont (2003), ^11^Noreña and Eggermont (2006), ^∗^ indicates behavioral tinnitus, • indicates PTS or TTS present.

## Do Animal Models of Tinnitus Relate to Tinnitus Findings in Humans?

The effects of tinnitus were until recently (Chen et al., 2014, 2015) studied very differently in animal models compared to humans. First of all detecting tinnitus is straightforward in humans—one just has to ask, whereas in animals it has to be inferred from behavioral tests. This is not straightforward (Eggermont, 2013; Lobarinas et al., 2013; Salloum et al., 2014, 2016), but let’s assume that it can be done unambiguously. Secondly, putative electrophysiological correlates of tinnitus in animal models are increased SFRs, increased pair-wise spike-firing synchrony, and changes in the tonotopic maps in the auditory system (Eggermont and Roberts, 2004; Eggermont, 2012). In human studies one finds reduced or increased power of certain brain rhythms, interpreted as increased neural synchrony (Weisz et al., 2011; Weisz and Obleser, 2014), and changes in connectivity between brain areas based on EEG or functional magnetic resonance imaging (fMRI; Vanneste et al., 2011; Husain and Schmidt, 2014). Here, it is important to distinguish spike-firing synchrony and neural synchrony. I used spike firing synchrony as correlated firing times between two simultaneously recorded neurons. I use neural synchrony as in phase responding of population responses, typical EEG/MEG or slow BOLD fluctuations, at two brain sites.

Humans potentially may show changes in tonotopic maps but these will be more likely related to hearing loss than to tinnitus (Langers et al., 2012). More indirect correlates of tinnitus can be deduced from stimulus-evoked activity (Gu et al., 2010; Roberts et al., 2010, 2013) but are more sensitive to co-occurring hyperacusis.

## Tinnitus Networks

### Putative Networks in Humans

Tinnitus may be related to changes in the resting-state neural networks of the brain. In a recent meta analysis of reported neural network changes in tinnitus patients, Husain and Schmidt (2014) found changes in the default network, in the connectivity between ACx and the limbic system that mediates stress, in the connection of the auditory system with the limbic system and attention network, and also in connections between visual cortex and the ACx, and between visual cortex and the attention network (Roberts et al., 2013). In contrast, Davies et al. (2014) did not find “significant differences in auditory network connectivity between groups after correcting for multiple statistical comparisons in the analysis. This contradicts previous findings reporting reduced auditory network connectivity; albeit at a less stringent statistical [significance] threshold.”

**FIGURE 2:**
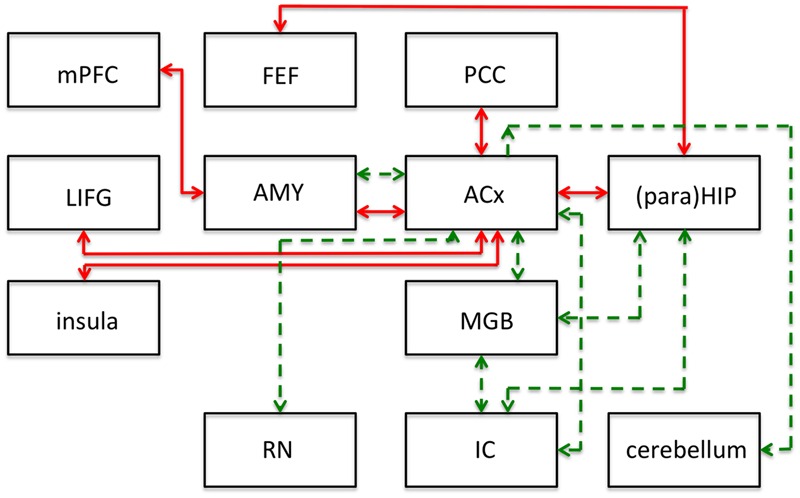
**Summary of main results of resting-state functional connectivity studies in tinnitus in humans (red lines) and following salicylate application in rats (green lines).** This figure shows modifications to the connections of the networks and does not represent the networks in their entirety. ACx, auditory cortex; AMY, amygdala; FEF, frontal eye fields; IC, inferior colliculus; LIFG, left inferior frontal gyrus; MGB, medial geniculate body; mPFC, medial prefrontal cortex; PCC, posterior cingulate cortex; (para)HIP, parahippocampus and hippocampus. Based on human data from Husain and Schmidt (2014), and animal data from Chen et al. (2015).

Non-auditory areas have been identified as involved in people with tinnitus, using non-invasive functional and structural imaging. Resting state connectivity between brain areas is, by definition, based on spontaneous fluctuations in brain activity that can be reliably organized into coherent networks. The term “resting state” differentiates this type of activity from that obtained as a result of some task or stimulus (Husain and Schmidt, 2014). The finding of several resting state networks allows studying the neural mechanisms of tinnitus or auditory processing in general. See **Figure 2** for a representative set of these networks, the human network connectivities are indicated in red. It should be emphasized that the first insights into the role of inherent long-range cortical coupling in tinnitus were provided by resting-state studies probed by MEG (e.g., Weisz et al., 2007) and EEG (Vanneste et al., 2011).

### A Salicylate-Activated Tinnitus Network

To identify putative neural substrates for tinnitus and hyperacusis in an animal model, Chen et al. (2015) applied salicylate to rats and used behavioral, electrophysiological, and fMRI (7T animal MRI scanner) techniques to identify a putative tinnitus–hyperacusis network. They found that salicylate application depressed the neural output of the cochlea, as measured by the compound action potential. In contrast, strongly amplified sound-evoked local field potentials (LFPs) were obtained in the amygdala (AMY), MGB, and ACx. These findings relate in principle to central gain changes and potentially to hyperacusis. Resting-state fMRI, which may be more relevant to understand tinnitus, showed a hyperactive auditory network composed of IC, MGB, and ACx. This network was also connected to parts of the cerebellum, AMY, and reticular formation (RN; **Figure 2**; dashed green lines).

The connectivity analysis was done by seeding various voxels in the regions-of-interest. This basically shows one-way connectivity from the seed region to other areas, by combining the findings from various seed regions a putative network can be built up. When the IC was seeded, they found that activity changes in the IC correlated significantly with that in voxels of the MGB, interpreted as an increase in functional connectivity (FC; **Figure 2**). Similarly, when changes in MGB voxels showed increased FC with voxels in the ACx. With the seed in the ACx, increased FC was seen in the same two lower auditory centers, the MGB and IC, which suggests a recurrent feedback loop in this auditory subnetwork (IC, MGB, and ACx in **Figure 2**). FC further revealed enhanced coupling between the ACx and the cerebellum, the reticular nuclei, and the AMY, and between the IC, MGB, and hippocampus. These subdivisions all show large salicylate-induced increases in the amplitude of low-frequency fluctuations, as well as increased FC with the ACx.

Comparing the animal (green dashed lines in **Figure 2**) and human networks (red full lines) does not tell us too much; the only correspondence is in the connection between ACx and AMY, and the involvement of the hippocampus and the area surrounding it, the parahippocampus. The animal model emphasizes the strengthening of the connections of the auditory structures and the relevance of subcortical structures such as the reticular activating system and parts of the cerebellum. The human network in particular adds the involvement of the attention network (frontal eye fields, left inferior frontal gyrus, insula).

In this comparison, one should note that the human network covers tinnitus in humans regardless of its etiology, whereas the animal network is limited to the putative effects of salicylate: tinnitus as well as hyperacusis.

### Tinnitus without Hearing Loss

If one believes that increased SFR in the auditory nervous system, and particularly in ACx, is a neural correlate of tinnitus (Eggermont and Roberts, 2004; Roberts et al., 2010; Basura et al., 2015), then a few additional noise-exposure effects demand attention. After a single TTS-causing exposure—which constitutes the bulk of current animal experiments involving gap-startle indications of tinnitus—one often finds increased SFRs and the gap-startle reflex indicates (Salloum et al., 2014, 2016) the presence of tinnitus. Even more intriguing is that after long-term exposure (≥6 weeks) to 4–20 kHz sound (noise or multi-tone) with levels ≤80 dB SPL one finds in ACx that the exposure frequency range causes strong suppression of driven and spontaneous firing rates, whereas the edge regions (extending about one octave above and two octaves below the band-pass exposure range) show increased gain for sound stimuli and also increased SFR and increased neural synchrony (Noreña et al., 2006; Pienkowski and Eggermont, 2009; Munguia et al., 2013).

It is instructive to look at several cases with relatively low-level noise exposures in some more detail. Brozoski et al. (2002) behaviorally trained and tested chinchillas before and after unilateral exposure to a unilateral 80 dB SPL 4 kHz tone for 30–60 min. This elevated the ABR thresholds by 20–30 dB. In comparison to a non-exposed control group, they found that putative fusiform cells of exposed animals showed significantly elevated spontaneous activity. Compared with cells of unexposed animals, the exposed group displayed enhanced discrimination of 1 kHz tones and putative fusiform cells of exposed animals showed a greater stimulus-evoked response to tones at 1 kHz and at characteristic-frequency. This fits with the enhanced sound responses two octaves below our long-term 4–20 kHz exposure (Noreña et al., 2006). These are potential correlates of hyperacusis.

Noreña et al. (2006) continuously exposed four adult cats in their free-running room so that there was no time relationship with the feeding and cleaning period of about 0.5 h/day. More than 4 months exposure of these normal hearing adult cats with a 4–20 kHz band of multi-frequency tone pips—termed an enhanced acoustic environment (EAE)—continuously presented at 80 dB SPL, did not result in changes in ABR thresholds. However, there was a strong reduction in the driven firing rates to frequencies between 4 and 20 kHz, and an increase for frequencies below or above that range. The mean SFRs for CFs in the exposure frequency range was not significantly changed compared to controls, but the SFRs were significantly increased for units with CFs below and above the exposure frequencies. The similarity between the increases for the SFR and driven firing rate suggests an underlying synaptic gain change as the main cause. Neural synchrony was vastly increased as well, particularly when involving units with CFs above and below the exposure frequency range. Tonotopic maps were reorganized with CFs > 20 kHz taking over the normal 4–20 kHz CF range (Noreña et al., 2006).

We followed this up with several studies where the 4–20 kHz sound was presented at 68 dB SPL, and only for about 6 weeks. In our first study (Pienkowski and Eggermont, 2009), we reported basically the same pattern as in the Noreña et al.’s (2006) study. ABR thresholds were completely normal and so were DPOAEs. Tonotopic maps were reorganized, a process that surprisingly started during the 3-month recovery period in quiet (Pienkowski and Eggermont, 2009). Munguia et al. (2013) reported that for the 4–20 kHz multi-tone EAE, the SFR for MUs with CFs in the EAE range was significantly smaller than for those with CFs outside the EAE frequency region. In addition, the SFR for MUs with CFs outside the EAE frequency range (non-EAE) was significantly larger than for controls in the same frequency range. The increases in SFR were most often observed on the high-frequency side of the EAE. For instance, for the 4–20 kHz EAE, the mean ratios of the SFRs in exposed to control cats were 0.91 (below EAE range), 0.39 (within EAE range), and 1.47 (above EAE range).

An overview of some of these findings, augmented with results from Basura et al. (2015) and Wu et al. (2016) that are likely TTS causing, is presented in **Table 4**. Again, assuming that increased SFRs in ACx suggest the presence of tinnitus, one has to come to the conclusion that tinnitus cannot only occur in humans with clinical normal thresholds (≤25 dB HL) but also with absolute normal thresholds (Gu et al., 2010; Melcher et al., 2013). It should be noted that tonotopic map changes are not a requisite for tinnitus in humans with clinically normal audiograms (Langers et al., 2012), whereas the equivalent in noise-exposed animal suggests that tonotopic map changes do not occur for hearing losses <25 dB, whereas increased SFR may still be present (Seki and Eggermont, 2002, 2003).

**Table 4:** Effects of non-traumatic noise exposure.

Structure	Exposure level (SPL)	SFR	Tonotopic map	Synchrony	GABA	Tinnitus
ANF	96 dB; 5 days	≈^1^				
VCN	80, 103 dB; 2 h				⇑^2^	
DCN	80, 103 dB; 2 h				⇑^2^	
	80 dB, 30–60 min	⇑^3^				Yes
	97 dB; 2 h	⇑^4^		⇑^4^		Yes
IC	120 dB; 4 h	⇑^5^				
MGB						
A1	80 dB; ≥4 months	⇑^6^	Changed^6^	⇑^6^		
	68 dB; ∼6 weeks	⇑^7^	Changed^8^	⇑^8^		Yes
	97 dB; 2 h	⇑^9^				

*^1^Salvi et al. (1983), ^2^Idrizbegovic et al. (1998), ^3^Brozoski et al. (2002), ^4^Wu et al. (2016), ^5^Wang et al. (2013), ^6^Noreña et al. (2006), ^7^Munguia et al. (2013); ^8^Pienkowski and Eggermont (2009), ^9^Basura et al. (2015).*

## Making Animal Models and Human Tinnitus Research More Compatible

It is obvious that making the research approach between animal models and humans more comparable would require that animal recordings of neural activity include spontaneous LFPs, study the power in the various EEG frequency bands (delta, theta, alpha, beta, and gamma), and use simultaneous recordings in several auditory and non-auditory areas to assess changes in connectivity (Weisz and Obleser, 2014). This will require recording from awake animals.

Recently, Salvi and colleagues have made a start on this by recording LFPs and carrying out resting state and connectivity (fMRI) recordings in anesthetized rats (Chen et al., 2014, 2015). Human research using neural spiking activity can only be done in pre-surgical conditions such as for relief of epilepsy, but so far only depth-recorded LFPs are have been obtained (Sedley et al., 2015).

Thus, the large differences in what is recorded in animal models with those obtained in humans makes a direct approach to the heterogeneity of tinnitus difficult. The most human-compatible animal model currently is that from Chen et al. (2015), albeit that it is based on salicylate-induced tinnitus, and that provides for only a minute fraction of the etiology of tinnitus in humans.

## Conclusion

In humans with tinnitus, several biomarkers for tinnitus have been proposed based on spontaneous brain rhythms, both decreased and increased power in several frequency bands, and largely increased neural network connectivity between auditory and attention as well as limbic networks. In animal models, tinnitus biomarkers—increased SFR, burst-firing, and neural synchrony—are the same for acute noise trauma, chronic effects with permanent threshold shifts after recovery from trauma, but also for long-term non-traumatic exposure without hearing loss as measured by ABR, and normal DPOAEs. All the noise-exposure animal models reviewed here show signs of increased central gain (hyperacusis?) and increased SFR (tinnitus?). Salicylate application in animals, chronic as well as acute, despite causing a mild hearing loss, has different electrophysiological characteristics compared to chronic noise, both in periphery and in the cortex. Salicylate animals showed behavioral signs of hyperacusis as well as tinnitus, whereas the electrophysiological signs reflected increased central gain but no change in SFRs. Both noise exposure and salicylate application may cause tinnitus and hyperacusis-like effects, but differ in their effects on SFR. This is an illustration of heterogeneity in electrophysiological correlates of tinnitus for these two etiologies.

## Author Contributions

The author confirms being the sole contributor of this work and approved it for publication.

## Conflict of Interest Statement

The author declares that the research was conducted in the absence of any commercial or financial relationships that could be construed as a potential conflict of interest.
